# La campaña de erradicación de la malaria en Colombia, 1959-1979

**DOI:** 10.7705/biomedica.6250

**Published:** 2022-06-01

**Authors:** Julio César Padilla-Rodríguez, Mario Javier Olivera, Pablo Chaparro, Martha Lucía Quiñónez, José Pablo Escobar, Gilberto Álvarez

**Affiliations:** 1 Red de Gestión de Conocimiento, Investigación e Innovación en Malaria, Bogotá, D.C., Colombia Red de Gestión de Conocimiento, Investigación e Innovación en Malaria Bogotá, D.C. Colombia; 2 Grupo de Parasitología, Instituto Nacional de Salud, Bogotá, D.C., Colombia Instituto Nacional de Salud Bogotá, D.C. Colombia; 3 Observatorio Nacional de Salud, Instituto Nacional de Salud, Bogotá, D.C., Colombia Instituto Nacional de Salud Bogotá, D.C. Colombia; 4 Departamento de Salud Pública, Facultad de Medicina, Universidad Nacional de Colombia, Bogotá, D.C., Colombia Universidad Nacional de Colombia Universidad Nacional de Colombia Bogotá, D.C. Colombia; 5 Facultad Nacional de Salud Pública, Universidad de Antioquia, Medellín, Colombia Universidad de Antioquia Universidad de Antioquia Medellín Colombia; 6 Secretaría Departamental de Salud de Cundinamarca, Bogotá, D.C., Colombia Secretaría Departamental de Salud de Cundinamarca Bogotá, D.C. Colombia

**Keywords:** malaria, erradicación de la enfermedad, epidemiología, control, historia, Colombia, Malaria, disease eradication, epidemiology, control, history, Colombia

## Abstract

**Introducción.:**

A mediados de la década de 1950, el país adoptó e implementó la Campaña de Erradicación de la Malaria (CEM), sin que hasta ahora se haya hecho su evaluación.

**Objetivo.:**

Evaluar los resultados alcanzados en las fases de ataque y consolidación de la campaña de erradicación de la malaria en Colombia, entre 1959 y 1979.

**Materiales y métodos.:**

Se hizo un estudio descriptivo y retrospectivo de los resultados “malariométricos” y operacionales de la CEM en Colombia entre 1959 y 1979 a partir de los datos recopilados de los archivos del Ministerio de Salud Pública. Se utilizaron los criterios establecidos por la Organización Mundial de la Salud (OMS) relacionados con las fases de un programa de erradicación de malaria. Se almacenó, tabuló y analizó la información, y se elaboraron y aplicaron indicadores malariométricos.

**Resultados.:**

En el periodo de erradicación a corto plazo (1959-1969), durante el primer año de la fase de ataque (1959), se alcanzó una reducción de la transmisión del 94 % (4.172) y, en el último año (1962), una disminución del 88 % (8.426) en la carga acumulada de casos comparada con el promedio anual de la década del 50 (71.031); estos bajos niveles de transmisión se mantuvieron hasta finales de 1969. En el periodo de intensificación del control para la erradicación (1970-1979), se produjo un incremento de la endemia y resurgió la transmisión epidémica. Debido a problemas financieros que afectaron la regularidad de la operación para mantener los resultados, y no habiéndose logrado la interrupción de la transmisión, se observó un resurgimiento de casos en las fases de ataque y consolidación.

**Conclusiones.:**

La campaña no logró la meta de interrupción de la transmisión de la malaria en el territorio nacional, pero sí se consiguió un acentuado control en áreas de mediana y baja intensidad.

## Abstract (en)

**Introduction:**

In the mid-fifties, Colombia adopted and implemented the Malaria Eradication Campaign (MEC), which has not been evaluated yet in the country.

**Objective:**

To evaluate the results achieved during the attack and consolidation phases of the MEC regarding malaria transmission in Colombia between 1959 and 1979.

**Materials and methods:**

We conducted a descriptive and retrospective study based on the malariometric and operational results of the MEC in Colombia from 1959 to 1979 compiled from the archives of the Ministry of Public Health. We used the criteria defined by the WHO Expert Committee on Malaria. The information was stored, tabulated, and analyzed based on the malariometric indicators we developed.

**Results:**

In the short-term eradication period (1959-1969), a reduction of 94% (4,172) in transmission was achieved during the first year of the attack phase (1959) while in the last year (1962), there was an 88% (8,426) reduction of the accumulated load of cases compared to the annual average of the 1950s (71,031). These low levels of transmission were maintained until the end of 1969. During the intensification period of control of the eradication (1970-1979), there was an increase in malaria endemicity and a resurgence of the epidemic transmission. Due to financial problems affecting the regularity of the operations, Colombia was unable to sustain the results and achieve an interruption of transmission, which resulted in a resurgence of cases during the attack and consolidation phases.

**Conclusions:**

The campaign did not achieve the goal of interrupting malaria transmission in the national territory but there was marked control in areas of medium and low intensity.

A comienzos de la segunda mitad del siglo XX se estimaba que el total de casos anuales de malaria a nivel mundial era de 250 a 350 millones y que ocurrían entre 2 y 5 millones de muertes por esta causa. La malaria se consideraba la enfermedad responsable de la mayor carga económica y social en la población de los trópicos ^1^^,^^2^. En la década de 1950 en Colombia, las proyecciones que se tenían sobre la magnitud del problema eran abrumadoras.

Durante la fase preparatoria de la Campaña de Erradicación de la Malaria (CEM), se estimó que el área afectada por la malaria en el territorio nacional era de 970.849 km^2^, es decir, el 83 % del país, con cerca de 600.000 casos anuales. A pesar del evidente subregistro, los organismos de salubridad notificaban anualmente 71.320 casos y unas 1.576 muertes por esta causa. Prevalecían las infecciones parasitarias producidas por *Plasmodium vivax* (60 %) y los vectores principales eran *Anopheles albimanus*, *A. nuñeztovari*, *A. darlingi* y *A. pseudopunctipennis*. Se calculaba que el impacto económico que producía la enfermedad en Colombia era de unos USD $ 30 millones al año ^3^^,^^4^.

Finalizada la Segunda Guerra Mundial, y con la emergencia de un nuevo orden político y económico global, algunas de las prioridades de la nueva potencia mundial eran los retos que implicaba el desarrollo de los países, el fortalecimiento e intensificación del comercio mundial, la producción de alimentos y el crecimiento de la población ^5^^,^^6^. En ese contexto nace la Organización Mundial de la Salud (OMS) como una de las entidades de la Organización de las Naciones Unidas (ONU), cuya misión esencial era la protección del comercio mundial, la facilitación de las actividades de explotación de recursos y la apertura de nuevos mercados ^7^^,^^8^. Por su impacto económico y el obstáculo que había representado para el comercio mundial, la malaria fue una de las enfermedades priorizadas. Así, la OMS entró a ejercer un papel fundamental para liderar, impulsar e implementar una de las iniciativas políticas más ambiciosas en salud pública desplegada hasta ese momento a nivel mundial, la “Campaña mundial de erradicación de la malaria” ^9^^-^^11^.

Entre los principales argumentos que justificaron la implementación de la campaña de erradicación de la malaria se destacan la importancia económica y la elevada carga social que representaba la malaria; los avances en el conocimiento científico sobre el tema y la tecnología disponible; las experiencias exitosas de control y la protección de poblaciones civiles y militares durante la Segunda Guerra Mundial; la existencia de instituciones de lucha contra la malaria en la mayoría de los países; el supuesto ahorro a largo plazo que supondría la erradicación frente al costo continuo de las medidas de control, y, por último, la necesidad de iniciar inmediatamente la campaña dada la esperada aparición de resistencia vectorial al dicloro-difenil-tricloetano (DDT) ^12^^,^^13^.

Durante la XIV Conferencia Sanitaria Panamericana celebrada en octubre de 1954 en Santiago (Chile), los países endémicos para malaria en las Américas adoptaron un plan continental para la erradicación de la enfermedad ^14^. En 1955, en el marco de la Octava Asamblea Mundial de la Salud celebrada en México, se aprobó la política de erradicación de la malaria y se acordó que la OMS lideraría la iniciativa, brindando asesoría técnica y apoyando la reorientación urgente de los programas de control de la enfermedad hacia planes de erradicación, y fomentando la investigación, la obtención de contribuciones financieras y la coordinación de los recursos para la ejecución del programa de erradicación en los países comprometidos ^11^, todo ello a cargo de un comité de expertos en malaria de la OMS.

En su sexto informe, este comité definió la erradicación como: “[...] la supresión de la transmisión de la enfermedad y del reservorio de casos infecciosos mediante una campaña de tiempo limitado llevada con tal perfección que, cuando acabe, no se restablezca la transmisión [.]” ^15^, y estableció cuatro fases para dicha erradicación: preparación, ataque, consolidación y mantenimiento, con una duración de cinco a ocho años en total. La fase preparatoria pretendía delimitar el área afectada por la malaria, los niveles de endemicidad y la intensidad de la transmisión estacional en las áreas endémicas, así como la preparación y organización de la respuesta, la planeación y las operaciones preliminares ^15^.

En Colombia, esta fase se llevó a cabo entre 1954 y 1956, y estuvo a cargo de la División de Malariología y la Organización Sanitaria Panamericana (OSP). Se logró delimitar el área objeto de las operaciones de la campaña y definir un plan racional para abocarlas. Se pudo establecer que el área palúdica del país representaba el 83 % (970.849 km^2^) de su superficie, con una población en riesgo de 9’304.397 habitantes (65 % de la población total). Se entrenó al personal operativo y técnico, y se elaboraron las normas y procedimientos operativos. En 1956, se creó el Servicio de Erradicación de la Malaria y se establecieron 16 zonas operativas en el territorio nacional, así como un plan de adquisición de insumos críticos, materiales, la infraestructura tecnológica y logística requerida, y pruebas piloto con insecticidas, evaluación epidemiológica y administración de las zonas. Además, el equipo de investigaciones trabajó en un censo de población y vivienda en el área afectada por la enfermedad ^16^^,^^17^.

Durante la fase de ataque de la campaña, se realizaron rociamientos intradomiciliarios con el insecticida DDT en todas las viviendas ubicadas en las áreas con malaria, y hubo vigilancia y evaluación de las actividades operativas para mantener la interrupción de la transmisión durante el tiempo necesario ^15^. El plan de erradicación contempló ocho ciclos de rociamientos intradomiciliarios semestrales con DDT, con una cobertura total en 1959 (A_1_), 1960 (A_2_), 1961(A_3_) y 1962 (A_4_). En este último año, se tenía previsto mantener el rociamiento intradomiciliario en toda el área durante el primer semestre y, en el segundo, en áreas de alta vulnerabilidad seleccionadas. Al finalizar ese año, culminarían todas las operaciones de campo para entrar en la fase de vigilancia. Cuando se evidenciara la reducción o interrupción de la transmisión, se suspendería el rociado y se iniciaría la fase de consolidación. Alcanzada la meta de erradicación, se pasaría a la fase de mantenimiento a cargo de los servicios de salud pública ^17^^,^^18^.

A pesar de que la CEM se implementó en el territorio nacional siguiendo estrictamente las recomendaciones técnicas del comité de expertos de la OMS, y se contó con la asistencia, vigilancia y evaluación técnica de la OSP, solo se elaboraron algunos informes sobre el avance de la iniciativa en los países de las Américas. En Colombia, existen únicamente informes parciales sobre el desarrollo de las diferentes etapas de la iniciativa que no están sistematizados. Hasta ahora no se ha hecho una evaluación técnica objetiva de los resultados de la CEM en cuanto a la transmisión de la malaria en el país. Además, los responsables políticos de este tema no han tenido en cuenta las múltiples lecciones aprendidas en la ejecución de dicha campaña, lecciones que podrían contribuir a mejorar el enfoque del actual plan estratégico nacional de malaria y a fortalecerlo para garantizar el cumplimiento del renovado reto de eliminar la malaria en el país para el 2035.

En este contexto, el objetivo del presente estudio fue evaluar los resultados de las fases de ataque y consolidación de la CEM campaña de erradicación de la malaria (CEM)en cuanto al comportamiento epidemiológico de la malaria en el territorio nacional entre 1959 y 1979.

## Materiales y métodos

Se hizo un estudio observacional descriptivo y retrospectivo que evaluó los resultados de la CEM en el territorio nacional en el periodo de 1959 a 1979. Se consultaron las fuentes oficiales de información secundaria del Servicio Erradicación de la Malaria del Ministerio de Salud y la Dirección de Campañas Directas. Además, se revisaron los informes de evaluación de los programas de malaria realizados por la OPS y se utilizó la información oficial del Departamento Administrativo Nacional de Estadísticas (DANE) para estimar la población en riesgo. La información geográfica y climática del Instituto Geográfico Agustín Codazzi y del Instituto de Hidrología, Meteorología y Estudios Ambientales, se empleó para estimar las variables ambientales y geográficas correspondientes al periodo de estudio.

Se analizaron variables de persona (población en riesgo, casos generales y específicos de malaria), lugar (regiones ecoepidemiológicas, departamentos endémicos e intervenciones) y tiempo (años e intervenciones). Se adoptaron las regiones ecoepidemiológicas oficiales de transmisión de malaria: Urabá- Bajo Cauca-Sinú-San Jorge, Pacífico, Amazonía, Orinoquía, Caribe y Andina.

Para analizar la primera etapa (1959-1969), se utilizaron los diferentes criterios y directrices técnicas recomendadas para el desarrollo y la evaluación de las fases de ataque y consolidación de la CEM establecidos en el sexto informe del comité de expertos en malaria ^15^^,^^19^. Para la evaluación de la segunda fase (1970-1979), también se tuvieron en cuenta las modificaciones técnicas introducidas por dicho comité ^15^^,^^19^.

Se elaboró y validó una base de datos en Excel para almacenar y tabular los datos recolectados. Se construyeron las medidas de frecuencias absolutas de las muestras examinadas, las muestras positivas, los casos de malaria general y por regiones, los casos por especie parasitaria y lugar de origen de infección, así como el número de rociamientos intradomiciliarios con DDT. Además, se establecieron las medidas de frecuencia relativas del índice parasitario anual (IPA), definido como el número de casos nuevos de malaria confirmados por 1.000 individuos en riesgo en un año específico; la de *P. falciparum* (FRIF), es decir, el porcentaje de personas infectadas con *P. falciparum*; el índice anual de *P. vivax* (IVA), que expresa la relación del número de casos de *P. vivax* dividido por 1.000 individuos en riesgo; el índice anual de sangre examinada (IASE), es decir, el número de láminas examinadas dividido por la población en riesgo sometida a vigilancia por 100, y el índice de láminas positivas (ILP), el cual indica la relación entre el número de láminas positivas y el total de láminas examinadas por 100. Se aplicaron medidas estadísticas de resumen en promedios, y de posición en medianas y valores extremos. Se hizo el análisis de tendencias del comportamiento de la malaria en Colombia en el periodo de estudio, y se elaboraron cuadros consolidados con las diferentes variables generales y por regiones.

Se acogieron los requisitos éticos establecidos en la Resolución 8430 de 1993 del Ministerio de Salud de Colombia, en cuyo artículo 11 se establece que estudios como el presente son investigaciones sin riesgo y no requieren aprobación de los comités de ética. Se garantizó la confidencialidad y anonimato de los datos.

## Resultados

La ejecución de la CEM en Colombia en el periodo 1959-1979 comprendió una fase inicial de erradicación a corto plazo, entre 1959 y 1969, y otra de intensificación del control con fines de erradicación, entre 1970 y 1979.

### Fase de ataque y consolidación, 1959-1969

La fase de ataque de la CEM en el país se programó a cuatros años (19591962). Incluía una etapa inicial que se desarrolló durante 1959 (A_1_) y 1960 (A_2_), y una final, implementada durante 1961 (A_3_) y 1962 (A_4_). Antes del inicio de la fase de ataque, se había establecido que el parámetro de referencia para comparar sería el promedio de casos anuales de malaria registrados en la década de 1950, lo que correspondía a 71.031 casos anuales.

**Figura 1:**
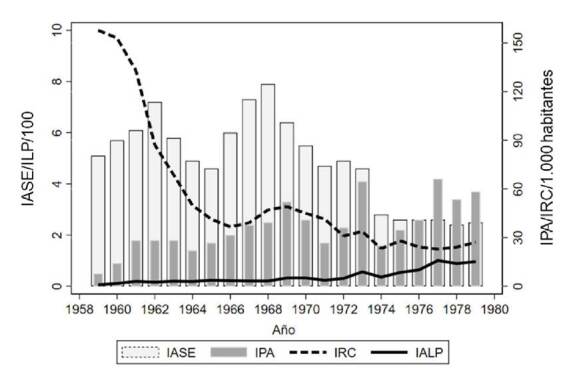
Indicadores malariométricos de la Campaña de Erradicación de la Malaria en Colombia, 1959-1979

IASE: índice anual de sangre examinada; IPA: índice parasitario anual; IRC: índice de rociamiento de casas; IALP: índice anual de láminas positivas

El comportamiento epidemiológico entre 1959 y 1962 evidenció la mayor reducción de la endemia durante la fase de ataque inicial, pues se pasó de 71.031 casos a 4.172 en A_1_ (94 % de reducción) y a 8.426 en A_2_ (88 % de reducción). Al finalizar 1969, se registraron 38.953 casos, es decir, hubo una reducción del 45 % (disminución de 32.078 casos) (cuadro 1). En contraste, el índice parasitario anual de malaria aumentó de 0,9 por 1,000 en 1960 a 3,3 en 1969 (figura 1). En la década de 1970, se registró un promedio de 42.252 casos anuales, y, a pesar de que hubo una reducción de la transmisión del 42 % (disminución de 29.779) en relación con el promedio de referencia, se empezó a evidenciar un incremento progresivo en los niveles endémicos y la reaparición de brotes epidémicos en los años 1973, 1977 y 1979. El índice anual de sangre examinada mostró una tendencia descendente con valores que oscilaron entre 7,9 por 100 (1968) y 2,5 por 100 (1979) (cuadro 1).

**Cuadro 1:** Índices malariométricos de la Campaña de Erradicación de la Malaria en Colombia, 1959-1979

Años	Población en riesgo	N° muestras examinadas	Casos			N° RID	Índices malariométricos				
			Total	*P. falciparum*	*P. vivax*		IPA	IASE	IALP	FRIF	IRC
1959	8’876.900	449.800	4.172	1.460	2.712	2’357.627	0,5	5,1	0,9	35,0	157,8
1960	9’018.368	509.920	8.426	3.595	4.831	2’358.989	0,9	5,7	1,7	42,7	153, 02
1961	9’316.611	570.160	16.974	9.928	7.046	2’127.057	1,8	6,1	3,0	58,5	133,02
1962	9’624.615	697.245	17.497	9.408	8.089	1’431.774	1,8	7,2	2,5	53,8	87,21
1963	9’942.999	577.406	17.898	9.074	8.824	1’163.280	1,8	5,8	3,1	50,7	68,67
1964	10’270.154	499.634	14.728	8.440	6.288	871.294	1,4	4,9	2,9	57,3	49,83
1965	10’610.404	488.366	18.240	11.575	6.665	744.002	1,7	4,6	3,7	63,5	41,34
1966	10’949.390	655.897	22.148	12.206	9.942	677.228	2,0	6,0	3,4	55,1	36,67
1967	11’299.223	824.669	26.570	15.044	11.526	741.895	2,4	7,3	3,2	56,6	39,14
1968	10’859.263	857.439	27.286	15.602	11.684	916.892	2,5	7,9	3,2	57,2	47,11
1969	11’794.673	760.073	38.953	23.225	15.728	980.578	3,3	6,4	5,1	59,6	49,07
1970	12’462.645	680.571	31.889	17.225	14.664	922.943	2,6	5,5	4,7	54,0	44,96
1971	12’873.225	600.204	22.206	11.451	10.755	873.910	1,7	4,7	3,7	51,6	41,44
1972	13’223.912	642.259	30.763	17.549	13.214	671.412	2,3	4,9	4,8	57,0	30,99
1973	13’537.950	628.057	56.119	33.892	22.227	754.124	4,1	4,6	8,9	60,4	33,75
1974	14’168.573	403.020	22.385	10.183	12.202	533.332	1,6	2,8	5,6	45,5	23,21
1975	14’542.280	384.364	32.572	15.556	17.006	663.863	2,2	2,6	8,5	47,8	28,08
1976	14’942.920	385.659	38.839	18.542	20.297	589.367	2,6	2,6	10,1	47,7	24,22
1977	15’354.597	401.621	63.888	29.753	34.135	573.765	4,2	2,6	15,9	46,6	22,91
1978	15’777.616	379.994	53.116	21.197	31.919	618.052	3,4	2,4	14,0	39,9	24,1
1979	16’212.289	399.478	60.738	23.184	37.554	714.348	3,7	2,5	15,2	38,2	27,1

RID: rociamientos intradomiciliarios; IPA: índice parasitario anual; IASE: índice anual de sangre examinada; IALP: índice anual de láminas positivas, FRIR: frecuencia relativa de *P. falciparum*; IRC: índice de rociamiento de casas

**Figura 2:**
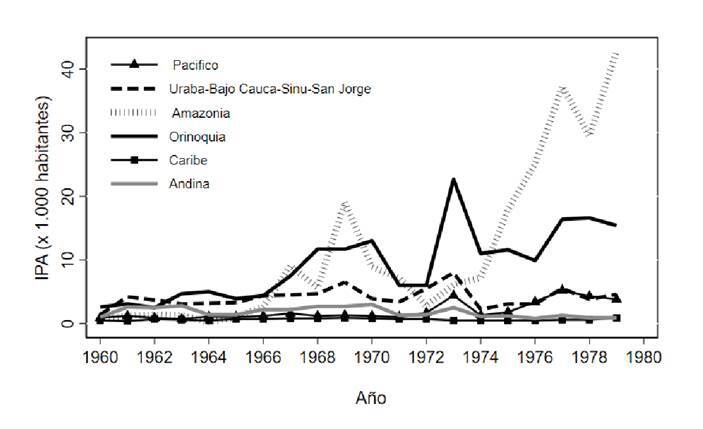
Comportamiento de la malaria por regiones ecoepidemiológicas durante la Campaña de Erradicación de la Malaria en Colombia, 1959-1979

Se llevaron a cabo las actividades operativas programadas durante el A_1_ y se intervinieron 1’178.814 viviendas (cobertura del 100 %) con rociamientos intradomiciliarios, usando el insecticida de acción residual DDT (grado técnico: 2 g/m^2^) en ciclos semestrales y en una extensión de 970.894 km^2^ (83 %) del área con malaria a nivel nacional. En 1959 (A_1_), se alcanzó la cobertura total del área, equivalente a 2’358.989 rociamientos intradomiciliarios, es decir, un índice de rociamiento de casas de 158 por 1.000 habitantes. La cobertura alcanzada se mantuvo durante 1960 (A_2_) con el tercer y cuarto ciclos de rociamientos intradomiciliarios programados en el área objeto de la campaña. Ese año el total fue de 2’357.627 rociamientos intradomiciliarios, equivalentes a un índice de rociamiento de casas de 153 por cada 1.000 habitantes, con lo que se completó la fase inicial (A_1_-A_2_) programada en el plan de erradicación de la malaria (cuadro 1).

Los resultados de la evaluación de la CEM por regiones ecoepidemiológicas en las dos décadas, evidenciaron una reducción de alrededor del 90 % de la endemia palúdica en casi todas las regiones, excepto en la Andina y la Caribe (figura 2). Al analizar los índices malariométricos básicos en A_1_, se encontró que la mediana del índice parasitario anual fue de 0,5 por 1.000 habitantes. Las regiones con mayor y menor valor de este índice fueron la Orinoquia (1,3 por 1.000 habitantes) y la Andina (0,3 por 1.000), respectivamente (cuadro 2). La región del Pacífico registró la mayor frecuencia relativa de infecciones por *P. falciparum* (62 %)(cuadro 2).

**Cuadro 2:** Índices malariométricos por regiones ecoepidemiológicas durante la fase de ataque en Colombia, 1959-1962

Regiones	Años	Población en riesgo	Muestras		Especie parasitaria		Índices malariométricos			
			Examinadas	*Positivas*	*P. falciparum*	P. vivax	IPA	FRIF	IASE	IALP
Urabá-Bajo	1959	1’559.109	76.687	1.150	345	805	0,7	30,0	4,9	1,5
Cauca -	1963	1’775.548	121.491	5.554	2.787	2.767	3,1	50,2	6,8	4,6
San Jorge-	1969	2’339.202	153.621	15.177	9.761	5.416	6,5	64,3	2,1	9,9
Sinú	1979	3’621.098	89.232	16.666	6.770	9.896	4,6	40,6	2,5	18,7
	Subtotal		364.344	37.397	19.318	18.079				
Pacífico	1959	1’820.685	131.154	820	451	369	0,5	0,3	7,2	0,6
	1963	2’073.027	117.020	1487	970	517	0,7	65,2	5,6	1,3
	1969	2’638.789	170.551	3.376	2.457	919	1,3	72,8	6,5	2,0
	1979	3’096.690	82.475	11.700	6.832	4.868	3,8	58,4	2,7	14,2
	Subtotal		370.046	16.563	10.259	6.304				
Amazonia	1959	174.953	9.933	68	20	48	0,4	29,4	5,7	0,7
	1963	199.193	13.333	254	34	220	1,3	13,4	6,7	1,9
	1969	233.046	34.708	4.442	3.182	1.260	19,1	71,6	5,0	12,8
	1979	377.642	52.222	16.181	3.798	12.383	42,8	23,5	13,8	31,0
	Subtotal		100.263	20.877	7.014	13.863				
Orinoquia	1959	212.475	6.563	282	158	124	1,3	56,0	3,1	4,3
	1963	241.921	22.659	1.146	709	437	4,7	61,9	9,4	5,1
	1969	249.832	29.920	2.912	1.520	1.392	11,7	52,2	12,0	9,7
	1979	551.211	39.169	8.489	2.980	5.509	15,4	35,1	7,1	21,7
	Subtotal		91.748	12.547	5.209	7.338				
Caribe	1959	2’549.704	75.023	1.206	543	663	0,5	45,0	2,9	1,6
	1963	2’917.037	115.167	1.721	1.090	631	0,6	63,3	3,9	1,5
	1969	3’281.416	185.100	3.080	1.794	1.286	0,9	58,2	0,0	1,7
	1979	4’421.336	68.049	3.972	1.832	2.140	0,9	46,1	1,5	5,8
	Subtotal		368.316	8.773	4.716	4.057				
Andina	1959	2’137.932	125.091	646	196	450	0,3	30,3	5,9	0,5
	1963	2’736.273	187.736	7.736	3.484	4.252	2,8	45,0	6,9	4,12
	1969	3’034.304	187.921	8.160	4.087	4.073	2,7	50,1	1,0	4,3
	1979	4’144.312	68.331	3.730	972	2.758	0,9	26,1	1,6	5,5
	Subtotal		443.988	19.626	8.543	11.083				
	Total		1’738.705	115.783	55.059	60.724				

IPA: índice parasitario anual; FRIR: frecuencia relativa de P. falciparum; IASE: índice anual de sangre examinada; IALP: índice anual de láminas positivas

En A_4_ (1962), se registró un acumulado de 17.497 casos confirmados de malaria. Se evidenció una reducción del 75 % (disminución de 53.534 casos) de la endemia con relación al promedio de los años 50. También, se observó un índice parasitario anual de malaria de 1,8 por 1.000 habitantes en riesgo; 7 de cada 100 personas en áreas de riesgo fueron sometidas a vigilancia epidemiológica, y 2,5 de cada 100 láminas examinadas fueron positivas para malaria (cuadro 1). Al finalizar 1969, cerca del 84 % (827.222 km^2^) del territorio originalmente delimitado se encontraba en la fase de ataque y se habían beneficiado 3’725.660 habitantes en áreas intervenidas por la CEM (cuadro 3).

**Cuadro 3:** Cobertura espacial y poblacional en las fases de ataque y consolidación de la Campaña de Erradicación de la Malaria en Colombia, 1963-1969

Años	Cobertura por superficie (km2)				Población beneficiada			
	Fase de ataque	%	Fase de consolidación	%	Fase de ataque	%	Fase de consolidación	%
1963	847.624	77,9	121.000	12,5	408.2017	44,2	5’200.000	56
1964	Sin actividades operativas	-	Sin actividades operativas	-	Sin actividades operativas	-	Sin actividades operativas	-
1965	304.343	71,5	276.294	28,1	2094309	21,1	7’829.286	79
1966	Sin actividades operativas	-	Sin actividades operativas	-	Sin actividades operativas	-	Sin actividades operativas	-
1967	710.347	72	251.929	25,6	700.000	7,5	8’127.223	87,7
1968	827.200	84	155.000	15,7	3.216.220	30,4	7’070.584	70
1969	827.222	84,1	11.7351	11,9	3.725.660	40,2	7’288.688	78,6

Al final de la década de los 60, se observó una reducción del 71 % de los niveles endémicos de la transmisión de la enfermedad con respecto a la línea de base (década de 1950), pues se pasó de un promedio de 71.031 casos anuales a 20.872. La mediana del índice parasitario anual de malaria en la década fue de 1,8 por 1.000 habitantes (valor máximo de 3,3 y valor mínimo de 0,9 por 1.000 habitantes); la proporción de P. falciparum fue, en promedio, de 57 %; de cada 100 muestras examinadas, en promedio, 3 eran positivas y la mediana del índice anual de sangre examinada fue de 6,1 % (valor máximo 7,9 y valor mínimo 4,6 %) (cuadro 1).

En 1963 (A_5_), se pasó a la etapa de consolidación en 121.000 km^2^ (12,5 %) del área original con malaria, con lo que se logró proteger a una población de 5,2 millones de habitantes (56 %) en riesgo. En 1964 y 1966 no se realizaron actividades operativas y en el siguiente 276.294 km^2^ aún se encontraban en fase de consolidación. Para 1967, solo se habían mantenido en consolidación 251.929 km^2^, y se había protegido a una población de 8’127.223 habitantes en las áreas de riesgo (cuadro 3). En 1969, el área consolidada era de 117.351 km^2^, el número de localidades varió muy poco y la población beneficiada se mantuvo en alrededor de 7’000.000 de personas protegidas (cuadro 3).

### Intensificación del control con el objetivo final de erradicación, 1970-1979

En los años 70, se focalizaron las acciones en municipios y localidades con una mayor concentración de casos. El promedio y la mediana de municipios endémicos positivos fue de 470. Los años con mayor y menor número de municipios positivos fueron 1977 (520) y 1971 (427). La mediana de localidades intervenidas fue de 4.503, con valores extremos que oscilaron entre 6.239 y 2.910. Se hicieron 6’640.845 rociamientos intradomiciliarios en el área programada durante el lapso, con una cobertura útil, en promedio, de 77,5 %. Se registró un acumulado de 412.515 casos de malaria en el país. Hubo un incremento del 97,6 % de la endemia comparado con lo observado en el periodo de 1960 a 1969. La mediana del índice parasitario anual de malaria en ese lapso fue de 2,6 por 1.000 habitantes (valor máximo 4,2 y valor mínimo de 1,6 por 1.000 habitantes). La malaria por *P. vivax* fue la de mayor prevalencia, 213.973 casos (52 %) (cuadro 4). Las regiones de Urabá- Bajo Cauca-San Jorge-Sinú y Pacífico contribuyeron con la mayor carga acumulada de casos, 216.734 (52,5 %), y fueron las de mayor incremento en la década de los 70 (cuadro 2).

**Cuadro 4:** Cobertura de actividades operativas en la Campaña de Erradicación de la Malaria en Colombia entre 1970 y 1979

Actividades operativas	1970	1971	1972	1973	1974	1975	1976	1977	1978	1979
Población	12’462.645	12’873.225	13’223.912	13’537.950	14’152.382	14’542.280	14’949.200	15’354.597	15’777.616	16’212.289
Casos confirmados	31.889	22.206	30.763	56.119	22.385	32.572	38.839	63.888	53.116	60.738
N° de municipios positivos	473	427	465	485	433	446	467	520	498	488
N° de localidades positivas	4.867	3.597	4.584	6.239	2.910	3.484	3.851	4.437	4.568	4.603
Viviendas para RID*	522.861	539.098	850.977	783.602	699.096	663.963	626.026	613.054	626.198	715.990
RID realizados	980.578	922.943	873.910	671.412	754124	533.332	663.863	589.367	572.765	618.052
% cobertura útil	88,1	84,0	85,2	79,2	79,0	76,9	75,0	71,0	72,2	76.7
IRC por 1.000	45	41,4	31,0	33,8	23,2	28,1	24,2	22,1	24,1	27,1

RID: rociamiento intradomiciliario; IRC: índice de rociamiento de casas

En la década de 1970, se evidenció que el índice anual de sangre examinada en la población en riesgo aumentó en todas las regiones, excepto en la región Andina. Igualmente, la positividad se incrementó en la mayoría de regiones, con el mayor aumento en la Orinoquia y en Urabá-Bajo Cauca-San Jorge-Sinú (cuadro 2). Hubo tres brotes epidémicos de malaria entre 1970 y 1979; el primero ocurrió en 1973, año en que se reportaron 56.119 casos y un índice parasitario anual de 51,4 por cada 1.000 habitantes en riesgo; el segundo en 1977, con el mayor número de casos, 63.888, y un índice parasitario anual de 90,5. El último brote de la década se reportó en 1979, cuando se registraron 60.738 casos y un índice parasitario anual de 94,4. Estas epidemias ocurrieron principalmente en municipios receptivos y de elevada vulnerabilidad en las regiones del Urabá-Bajo Cauca-San Jorge-Sinú, Pacífico y Amazonia (cuadro 2).

## Discusión

El estudio evidenció que la Campaña de Erradicación de la Malaria (CEM) implementada en Colombia entre 1959 y 1979, nunca logró alcanzar la meta de interrumpir la transmisión mediante la eliminación de los reservorios de la enfermedad en el territorio nacional. Sin embargo, durante la década de 1960 se logró una reducción histórica y significativa, aunque pasajera, en los niveles endémicos de la enfermedad, principalmente en las áreas receptivas con alta densidad poblacional pero con baja transmisión, y un aceptable control de la transmisión en áreas endémicas con gran potencial para la malaria. Estos resultados no fueron sostenibles en los años siguientes, por lo que en el resto del periodo resurgió la transmisión y hubo un franco deterioro de las medidas implementadas en toda el área endémica objeto de la campaña en el país.

Situaciones muy similares a la nuestra se observaron en algunos países de las Américas en que también se implementaba la estrategia de erradicación, como Brasil, Bolivia, Perú y Venezuela, donde era posible encontrar áreas que habían evolucionado favorablemente e, incluso, alcanzaron las fases de consolidación y mantenimiento, en tanto que en otras la evolución había sido menos favorable y se encontraban en fase de ataque ^20^^-^^22^. Estos resultados se pudieron evidenciar en el informe de la OSP sobre la situación de los programas de erradicación en la región de las Américas solicitado por la Asamblea Mundial de la Salud para establecer los avances de la iniciativa. Allí se documentaban los pocos resultados alcanzados y el estancamiento y los retrocesos en la mayoría de los países, y se pronosticaba que serían pocos los que lograrían la meta de erradicación de la enfermedad a corto plazo ^23^^-^^25^.

La OSP clasificó a los países según los avances logrados y la factibilidad de alcanzar la erradicación en todo su territorio o en parte. Colombia fue clasificada entre los países del grupo III, que se caracterizaban por ser unidades políticas con escasas perspectivas de supresión de la transmisión de la enfermedad y del reservorio de casos infecciosos de malaria en el corto plazo ^26^^,^^27^. En el ámbito nacional, el Servicio de Erradicación de la Malaria había logrado, en la etapa inicial de la campaña, una adecuada implementación de la fase de ataque, alcanzando resultados satisfactorios. Esto permitió el paso del 60 % del área intervenida a la fase de consolidación. Sin embargo, estos resultados no fueron sostenibles en el tiempo por problemas políticos, financieros, técnicos y administrativos ^28^^,^^29^.

A pesar de este panorama y de los resultados desalentadores de la CEM, la OMS reafirmó que el objetivo final de la política era lograr la erradicación completa de la enfermedad, sin fijar un plazo. Para ello, recomendó a los programas nacionales flexibilizar e introducir algunos cambios en el modelo. Se establecieron metas intermedias para reducir la incidencia y evitar situaciones epidémicas y muertes por malaria ^30^ que la Dirección de Campañas Directas del Ministerio de Salud de Colombia adoptó e incluyó en los planes operativos de los años setenta, flexibilizando el enfoque, y mejorando y optimizando el presupuesto de esta Dirección. Para garantizar la cobertura e intensificar las medidas de ataque, se fortalecieron la evaluación y la vigilancia en las áreas en fase de consolidación mediante la introducción de la terapia combinada contra la malaria y su tratamiento en masa, así como el uso de mezclas de insecticidas en áreas resistentes de transmisión persistente. Sin embargo, dichas modificaciones lograron poco impacto en la efectividad de la campaña ^31^.

En el contexto regional y en otras partes del mundo, se pronosticaba un inminente fracaso de la campaña, lo que suscitó discusiones sobre las probables causas. En su octavo informe, el comité de expertos de malaria de la OMS estableció que las fallas, deficiencias y dificultades de la campaña podían ser variadas y múltiples, y las clasificó en fallas técnicas, operacionales y administrativas ^32^^-^^34^. La Dirección de Campañas Directas del Ministerio de Salud de Colombia compartió y acató este concepto, afirmando que las causas del fracaso se debían, entre otros, a problemas técnicos como la resistencia de algunas cepas parasitarias de P. falciparum a la cloroquina, la exofilia de algunos vectores transmisores y la resistencia al DDT en algunas áreas endémicas. Además, señaló los problemas operacionales ocasionados por dificultades para garantizar la accesibilidad operativa a las áreas endémicas en regiones como el Pacífico y los territorios nacionales debido a restricciones presupuestarias y económicas, fallas en el adiestramiento, indisciplina e inestabilidad del personal de campo por la poca remuneración salarial y el recargo de horas laborales, las limitaciones en la disponibilidad oportuna de materiales, insumos críticos, equipos, y recursos operativos, y la obsolescencia de la infraestructura logística de apoyo para garantizar el cumplimiento oportuno de las actividades programadas.

Asimismo, resaltó factores contextuales determinantes como la intensa colonización y la migración poblacional de las áreas selváticas hacia focos de dispersión en el Magdalena Medio, el Bajo Cauca-San Jorge-Urabá, el Ariari, el alto Patía, Catatumbo y el alto Putumayo; la agudización de la situación de orden público en estas áreas, y el retiro de la financiación internacional aportada por la UNICEF y USAID. La baja operatividad y cobertura contribuyeron al incremento de la vulnerabilidad en áreas endémicas intervenidas, lo que propició la reinfección y la reaparición frecuente de brotes epidémicos en las áreas en fase de consolidación, que frecuentemente volvían a la fase de ataque haciendo insostenibles los logros alcanzados y ocasionando la transmisión persistente en áreas resistentes ^35^^-^^37^.

En oposición a estas consideraciones, reconocidos expertos nacionales e internacionales con un punto de vista objetivo y crítico plantearon que las verdaderas razones y explicaciones del fracaso de la CEM en los países endémicos de las Américas fueron mucho más estructurales, complejas y multidimensionales. Según ellos, la CEM hacía parte de una estrategia global de expansión, posicionamiento y dominación política, económica y cultural de la nueva potencia mundial en la posguerra, y de su inocultable interés en la explotación y comercialización de materias primas y capital humano barato en áreas tropicales endémicas para enfermedades transmisibles como la malaria y la fiebre amarilla.

Estas enfermedades constituían barreras que dificultaban la extracción de los recursos naturales y las materias primas, encareciendo las operaciones y produciendo elevadas pérdidas económicas que afectaban las inversiones de la emergente clase capitalista internacional y las grandes empresas transnacionales interesadas en esos lugares. Además, los expertos señalaban el interés militar de las conquistas o, por el contrario, las amenazas militares en los sitios de interés económico y la lucha contra la expansión del comunismo ^38^^-^^40^. Estas intenciones se veían matizadas por la aparente filantropía y las contribuciones de las fundaciones privadas con interés en la expansión de capitales en el extranjero y en obtener posiciones clave a través de organizaciones financieras, tratados internacionales y entidades globales de la salud como la OMS y la OPS ^41^^-^^43^.

Son múltiples los asuntos críticos que afectaron el desarrollo exitoso de la CEM. Fue una iniciativa ambiciosa y prescriptiva, diseñada con un alto nivel de improvisación, rígida, y que exigía resultados a corto plazo, lo cual generó enormes expectativas y falsas esperanzas. Los países endémicos la importaron, adoptaron y aplicaron de forma acrítica, siguiendo rigurosamente las recomendaciones técnicas establecidas por la OMS. Además, la pretendida conveniencia económica de la erradicación frente al control resultó ser, finalmente, una falacia bastante costosa ^44^^,^^45^.

El paradigma que sustentaba la campaña era biologicista, disonante y reduccionista, y negaba intencionalmente las realidades sociales y las condiciones de vida de las poblaciones afectadas en escenarios específicos y variados de transmisión ^46^^,^^47^. Se creyó que la solución del problema se podría alcanzar rápidamente con el nivel de conocimiento científico y tecnológico alcanzado y las experiencias exitosas de la Segunda Guerra Mundial en la protección de poblaciones civiles y militares con DDT y los nuevos antimaláricos sintéticos. Bajo el supuesto de garantizar resultados a corto plazo en forma efectiva, se concibió su ejecución como una campaña vertical conducida, asistida y apoyada técnicamente por expertos externos, y se le prestó poca atención al verdadero nivel de desarrollo de la capacidad de respuesta técnica y operativa local de los países. La participación de los actores sociales, sectoriales e institucionales involucrados en el problema no se tomó en serio, ignorando la posibilidad de su empoderamiento y su contribución a la sostenibilidad y respuesta oportuna del programa ^48^^,^^49^.

Muchas de las lecciones aprendidas en la CEM podrían tenerse en cuenta para mejorar la capacidad de gestión técnica y operativa en aras de enfrentar el reto de eliminación de la malaria en el 2035 planteado por las instituciones y autoridades sanitarias del país con base en el modelo de acción integral territorial y la ruta integral de atención de la malaria. Entre las lecciones aprendidas, se destacan la necesidad y obligatoriedad de lograr un verdadero compromiso, así como la voluntad política y el empoderamiento de todos los sectores institucionales y sociales involucrados en los niveles nacional, subnacional y local para que asuman responsabilidades estratégicas, tácticas y operativas específicas, garantizando que las políticas, planes, programas y proyectos respondan a la variabilidad y heterogeneidad de la malaria, y reorientando y fortaleciendo la gestión técnica operativa, y la capacidad de liderazgo y de conducción del programa de malaria en los diferentes niveles.

Los objetivos eran lograr el control oportuno y la sostenibilidad de las acciones preventivas y de promoción requeridas; estratificar, diferenciar y caracterizar los diferentes escenarios de transmisión en el territorio nacional; buscar el mejoramiento continuo y el fortalecimiento de la capacidad de gestión oportuna y eficiente de la respuesta técnica y operativa en el nivel local; fortalecer la infraestructura tecnológica, logística y operativa regional y local para consolidar un sistema de vigilancia integral que respalde la inteligencia epidemiológica; optimizar y mejorar la disponibilidad de recursos financieros y talento humano competente y la gestión de los insumos críticos; establecer programas de formación y capacitación continuada del talento humano ejecutivo, técnico y operativo, y brindar asistencia y apoyo técnico permanente del nivel nacional para vigilancia, evaluación y seguimiento de los planes, programas y proyectos ^49^.

Algunas dificultades para la realización del presente estudio fueron la obtención de la información a partir de diversas fuentes oficiales nacionales e internacionales, y su calidad y confiabilidad en la década de 1950, así como la necesidad de recalcular y unificar con precisión las poblaciones generales y específicas en riesgo, dado que la OPS recomendaba utilizar la población total como denominador, lo que diluía y sesgaba el análisis e interpretación de los indicadores de estudio. Es necesario desarrollar estudios sobre el tema para profundizar y comprender mejor la etapa de erradicación de la malaria en nuestro país.

En conclusión, la Campaña de Erradicación de la Malaria (CEM) en Colombia nunca logró alcanzar la anhelada meta de interrumpir la transmisión de la enfermedad en todo el territorio nacional, pero se logró una reducción importante en los niveles de transmisión en todas las regiones ecoepidemiológicas durante la primera etapa de la campaña, lo que no fue sostenible en la etapa final. El fracaso de la campaña no se debió únicamente a los problemas técnicos, operativos y administrativos del programa, sino esencialmente, a factores socioeconómicos, políticos y culturales estructurales, complejos, dinámicos y multidimensionales que nunca fueron considerados.
